# Pharmacokinetics of Lidocaine Hydrochloride Administered with or without Adrenaline for the Paravertebral Brachial Plexus Block in Dogs

**DOI:** 10.1371/journal.pone.0169745

**Published:** 2017-01-09

**Authors:** Amélie Choquette, Eric Troncy, Martin Guillot, France Varin, Jérôme R. E. del Castillo

**Affiliations:** 1 Québec’s Animal Pharmacology Research Group (GREPAQ), Department of Veterinary Biomedical Sciences, Université de Montréal, Saint-Hyacinthe, Québec, Canada; 2 Faculty of Pharmacy, Université de Montréal, Montréal, Québec, Canada; University of Bari, ITALY

## Abstract

Adrenaline is known to prolong the duration of local anesthesia but its effects on the pharmacokinetic processes of local anesthetic drugs are not fully understood. Our objective was to develop a compartmental model for quantification of adrenaline’s impact on the pharmacokinetics of perineurally-injected lidocaine in the dog. Dogs were subjected to paravertebral brachial plexus block using lidocaine alone or adrenalinated lidocaine. Data was collected through a prospective, randomised, blinded crossover protocol performed over three periods. Blood samples were collected during 180 minutes following block execution. Compartmental pharmacokinetic models were developed and their goodness-of-fit were compared. The lowering effects of adrenaline on the absorption of lidocaine were statistically determined with one-sided tests. A one-compartment disposition model with two successive zero-order absorption processes best fitted our experimental data. Adrenaline decreased the peak plasma lidocaine concentration by approximately 60% (*P <* 0.001), decreased this local anesthetic’s fast and slow zero-order absorption rates respectively by 50% and 90% (*P* = 0.046, and *P* < 0.001), which respective durations were prolonged by 90% and 1300% (*P* < 0.020 and *P* < 0.001). Lidocaine demonstrated a previously unreported atypical absorption profile following its paravertebral injection in dogs. Adrenaline decreased the absorption rate of lidocaine and prolonged the duration of its absorption.

## Introduction

The concept of multimodal and pre-emptive analgesia has popularized the use of local and regional anesthetic blocks in small animal veterinary practice [1] Newly implemented dosage regimens, drug combinations and block techniques improve pain management for a variety of medical or surgical conditions [2–4]. Yet, the range of desensitization techniques is much narrower for the canine forelimb than for the hindlimb. The subscapular approach to the brachial plexus block is efficacious at desensitizing the forelimb from elbow to toes, but its effect proximal to the elbow is unpredictable at best [5,6] By targeting the nerve roots as they exit the vertebral foramen, the original and the modified paravertebral blocks of the brachial plexus seem promising for anesthetising the shoulder and brachium [7], but both approaches give inconsistent results and warrant further investigation.

Lidocaine is used in a variety of local anesthetic procedures, both in human and veterinary medicine, and its pharmacokinetics has been studied in different species for over 40 years [8,9]. Its destiny after intravenous and extravascular administration has been studied with different formulations, dosages or anesthetic contexts [10–12]. Yet, we are unaware of a published study describing the pharmacokinetics of lidocaine following peripheral nerve block in dogs.

Furthermore, several local anesthetics are available in solutions containing adrenaline, which decreases the regional blood flow and the rate of lidocaine absorption at injection site, hence extending the duration of the exposure of the targeted nerves to effective anesthetic concentrations [13]. Still, the effects of adrenaline on the absorption of lidocaine have not been thoroughly characterized [14], at least for this particular technique.

Among the few publications describing the effect of adrenaline on the pharmacokinetics of extravascular-administered local anesthetics, one used an interesting model to quantify the effect of adrenaline on the absorption of levobupivacaine following caudal epidural administration in children: these investigators fitted the drug’s time-concentration data to a model with two distinct first-order absorption rate constants whose onsets were separated by a lag time [15]. We hypothesized that this model could (1) be applied to other amide-type local anesthetics and extravascular administration sites, including the brachial plexus, and (2) accurately measure the lowering effects of adrenaline on the absorption rate of lidocaine. To verify our hypotheses, we performed a pharmacokinetic study of lidocaine either alone or combined with adrenaline. Our objectives were: first, to compare the goodness-of-fit of the above-described dual-absorption compartmental model (alternative model) and of the basic one-compartment model with a single first-order absorption process (standard model) to the plasma lidocaine data of both drug formulations (*i*.*e*. plain and adrenalinated lidocaine). Second, to determine whether the model with the highest goodness-of-fit could be further improved, and third, to quantify the effect of adrenaline on the absorption pharmacokinetics of lidocaine by analyzing the time-concentration data of both drug formulations with the best model.

## Materials & Methods

The study protocol was reviewed and approved by the Faculté de Médecine Vétérinaire Bioethics Committee (CEUA) of the Université de Montréal (protocol #Rech-1574). Animals were housed and handled following the guidelines of the Canadian Council on Animal Care. At the end of the study, the animals were returned alive and healthy to the institutional animal colony.

### Animals

Eight healthy female beagles, 2 to 5 years old, and weighing 11 (1.3) [mean (SD)] kg were used. They belong to our institutional Teaching & Research dog colony, where they are housed together in facilities that are approved by the Canadian Council on Animal Care, and accustomed to human interaction and manipulation by the veterinary students of the Comité d’Éthique et Bien-Être des Animaux (CEBA). The students interacted with them and exercised them daily except on the days of experimentation. Initial health status was assessed with a physical examination and standard blood tests (hematocrit, total proteins, uremia). Prior to each anesthesia, dogs were re-examined, weighed and fasted for 12 hours with free access to water.

### Study design and procedures

Dogs were sedated with an intramuscular combination of 10 μg/kg medetomidine hydrochloride and 0.1 mg/kg butorphanol tartrate. A 30-mm 20-gauge cephalic intravenous catheter was placed in the right cephalic vein for drug administration and blood sampling. Anesthesia was then induced with propofol to effect (maximum dose of 4 mg/kg), followed by endotracheal intubation and maintenance with ≈2% isoflurane in oxygen, as targeted on the vaporizer. Once the anesthesia depth was stabilized according to clinical signs, dogs were positioned in right recumbency, with the left forelimb pulled far caudally to improve visualization of the cervical seventh (C7) transverse process and the head of the first rib. To minimize the variability in block execution, all injections were performed on the left forelimb by the same left-handed investigator (A.C.). The fur along the lateral aspect of the neck was clipped from the transverse processes of C4 to the mid-scapula and from the spinous processes to the jugular groove. The skin was disinfected with isopropanol, which was also used instead of gel to facilitate transducer-skin contact. Ultrasound imaging (M-Turbo Vet^®^; FUJIFILM SonoSite Inc., Bothell, WA), was performed with a 6–15 MHz linear probe (HFL 50X Linear Transducer; FUJIFILM SonoSite Inc., Bothell, WA), using color-flow Doppler.

The ultrasound approach of Bagshaw *et al*. [16] was used in order to position the insulated needle (Echostim facet tip, 21-gauge, 40 mm; Havel’s Inc., Cincinnati, OH, USA) as close as possible to the nerve roots of C6, C7, and the junction of C8 and T1. Great care was taken not to lacerate/puncture a vessel or the targeted nerve sheath. Once the needle in position, the electrical stimulation technique (Stimuplex^®^ Dig RC, Braun Medical Inc., Bethlehem, PA, USA) was combined to ultrasound visualisation in order to validate needle positioning through the characteristic motor responses for each root: shoulder rotation with C6, elbow flexion with C7 and carpus/digits flexion with C8-T1 [7]. Details of the guided loco-regional block and rationale for diluting the tested local anesthetic solutions are subject of a separate publication [17].

Dogs were randomly assigned to one of two treatment groups using a block balanced randomized crossover design. The randomization was controlled for age, gender and body weight. Treatments consisted of commercially available injectable solutions containing 20 mg/mL of lidocaine hydrochloride, either alone or combined with 0.01 mg/mL adrenaline. Both formulations were diluted with sterile physiological saline at a 1:1 volume ratio prior to administration. For every block, 2 mg/kg lidocaine was administered per nerve root, to a total dose of 6 mg/kg per dog. After the third injection, isoflurane administration was immediately stopped and atipamezole hydrochloride administered intramuscularly at a dose of 50 μg/kg. Dogs were oxygenated until extubation and warmed as needed until they were awoken and capable of standing.

At the request of the bioethical committee, the dogs were used in three periods with a four-week washout between the first two treatments, and two weeks between second and third treatments. All washout periods exceed by at least 295 times the average 0.88 h terminal half-life of lidocaine in dogs [18]. In order to assure blindness of the investigators responsible for block execution and data collection, a technical assistant (D. G.) was in charge of randomization protocol and solution preparation.

### Pharmacokinetics

#### Blood sampling

One mL of blood was drawn *via* the cephalic vein catheter before each blood sampling to remove its heparin lock. Then, 3 mL of blood were collected in heparin tubes at 5, 8, 12, 16, 20, 30, 45, 60, 80, 100, 130, and 180 minutes after the time of first injection of plain or adrenalinated lidocaine. A 3-mL blood sample was additionally drawn at approximately 2 minutes after each lidocaine injection to detect a possible variation in drug absorption rate across injection sites. The precise time of blood sampling was noted. The samples were kept on crushed ice for a maximum of 8 hours and then centrifuged at 1300 × *g* and 4°C for 10 minutes. Plasma was harvested and stored at -80°C pending analysis.

#### Sample processing and analysis

The plasma lidocaine concentrations were measured using an adaptation of a published liquid-liquid back-extraction procedure [19]. Briefly, 75 μL of internal standard bupivacaine 10 μg/mL solution (Sigma-Aldrich, Milwaukee, WI, USA) and 175 μL of sodium carbonate 10% were added to 0.5 mL of plasma. The extraction was completed by adding 3 mL of n-hexane:methylene (4:1, v:v) and agitating gently for 30 minutes. After centrifugation, organic layers were transferred and combined to 250 μL of sulfuric acid 50 mM for back extraction. The tubes were vortexed and centrifugated again, the organic phase was then aspirated to waste and residual solvent removed from the acid (SpeedVac™ Plus SC210A; Savant Instruments, Farmingdale, NY, USA). The residual volume was reconstituted in a mobile phase consisting in a 70:30 mixture of 70 mM sodium sulfate and acetonitrile, and injected into a HP1050 HPLC system (Hewlett Packard, Wilmington, DE, USA) fitted with a C_8_ reversed-phase separation column (particle size 5μm, 150 mm x 4.6 mm inner diameter; Phenomenex, Torrance, CA, USA), and an UV detector set at 205 nm. Peak integration was performed using the Chem Station version A.09.01 (Agilent Technologies, Santa Clara, CA, USA). The mobile phase was prepared daily, injected at a 1.5 mL/min flow rate and maintained at 40°C. The analyte was spiked into a pool of drug-free plasma (4 mg/L) that was further diluted to yield standards at various concentrations down to the lowest limit of quantification (LLOQ) of 0.03125 mg/L.

The inter-assay precision and accuracy were 7% and 96.8%, respectively. The intra-assay accuracy was 97.1%, as assessed at two quality control concentrations (0.200 and 0.800 mg/L). The back-calculated concentrations were determined from the calibration curve of the day.

#### Pharmacokinetic analysis

The plasma concentration *versus* time data was subjected first to an exploratory non-compartmental analysis. The maximum plasma concentration (C_max_) and time to maximum concentration (t_max_) were determined directly from the data, and the total area under the curve (AUC) was calculated using the “linear-up / log-down” trapezoidal method. The terminal rate constant (λ_z_), the AUC extrapolation to infinity, and the apparent total clearance (CL/F) and volume of distribution (V/F) were estimated using standard formulae. Then, the estimated CL/F and V/F were used as initial parameter values in the population PK analysis using the ADAPT 5 software (ADAPT 5; Biomedical Simulations Resource, USC, San Diego, CA, USA).

**Fig 1:**
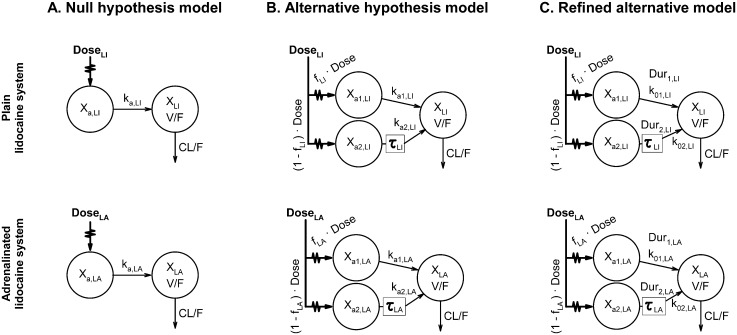
Compartmental pharmacokinetic models used for the analysis of paravertebrally injected lidocaine in dogs. Each model comprises one system for each of the two tested lidocaine formulations. (A) Standard model, with a single first-order absorption rate per system. (B) Alternative model, with two asynchronous first-order absorption rates per system. (C) Refined alternative model, with two time-constrained, asynchronous zero-order absorption rates per system. CL/F, apparent total clearance; Dose_j_, total amount of lidocaine administered (hereafter, subscript j = “LI” codes for the plain lidocaine formulation, and j = “LA” the adrenalinated lidocaine formulation); Dur_1,j_, duration of zero-order absorption from the first (early) absorption compartment; Dur_2,j_, duration of zero-order absorption from the second (late) absorption compartment; f_j_, dose fraction entering the first (early) absorption compartment; k_a,j_, first-order absorption rate constant; k_a1,j_, first-order absorption rate constant of the first (early) absorption compartment; k_a2,j_, first-order absorption rate constant of the second (late) absorption compartment; k_01,j_, zero-order absorption rate of the first (early) absorption compartment; k_02,j_, zero-order absorption rate of the second (late) absorption compartment; V/F, apparent distribution volume; **τ**_i_, lag-time of the second (late) absorption process; X_j_, amount of drug present in the disposition compartment; X_a,j_, amount of drug present in the absorption compartment; X_a1,j_, amount of drug in the first (early) absorption compartment; X_a2,j_, amount of drug in the second (late) absorption compartment.

To achieve our first objective, two sets of differential equations were developed. The first set (Model A–“null hypothesis”) comprised a pair of one-compartment disposition systems, one per drug formulation coded with subscript j, with common CL/F and V/F and a single first-order absorption process (k_a,j_) per system (Fig 1A). The second set (Model B–“alternative hypothesis”) was identical to Model A, except that each compartmental system had two first-order absorption processes (k_a1,j_ and k_a2,j_), of which k_a2,j_ was regulated by a lag time (τ_j_). In addition, a partition parameter (f_j_) estimated the dose fractions absorbed by each uptake process (Fig 1B).

To achieve our second objective, the residual analysis of Models A and B revealed that Model B could be refined into Model C (Fig 1C) by replacing the k_a1,j_ and k_a2,j_ of each compartmental system with two zero-order absorption processes (k_01,j_ and k_02,j_) that are active only for a specific duration (respectively, Dur_1,j_ and Dur_2,j_).

To fulfill our third objective, the data of both treatments (plain and adrenalinated lidocaine) was analyzed simultaneously and compared using all three pharmacokinetic models. The models were solved with maximum likelihood expectation-maximization methods (MLEM) using 3000 importance samples per iteration [20], assuming log-normally distributed pharmacokinetic parameters, and a diagonal covariance matrix of estimable parameters. Model selection was based both on the value of the Akaike goodness of fit information criterion (AIC) [21], and by comparing the scattering of standardized regression residuals [20].

#### Statistical analysis

Statistical analysis was conducted using SAS^®^ (SAS 9.3 Version; SAS Institute Inc., Cary, NC, USA). Descriptive statistics were used to assess group demographics, and values reported as percentages or mean (standard deviation, or SD). The estimated absorption kinetic parameters of the compartmental analysis, as well as the area under the plasma concentration curve between the time of dosing and the time of last measurable plasma concentration AUC_0-tlast,j_, C_max,j_ and t_max,j_, were compared at the α = 0.05 significance level using one-sided Student’s t tests on the raw or log-transformed values (*e*.*g*. for ln(k_0i_), H_0_: μ(adrenalinated lidocaine) ≥ μ(plain lidocaine), and for ln(Dur_i_), H_0_: μ(adrenalinated lidocaine) ≤ μ(plain lidocaine), in compliance with the hypothesized lowering effects of adrenaline on the absorption rate of lidocaine).

## Results

The crossover study was completed within a 3-month span. Seven dogs were available for the first period, and eight dogs for the second: therefore, we performed a 3^rd^ period with the missing dog plus three others (1 adrenalinated lidocaine, 2 plain lidocaine) that already completed the cross-over. In consequence, ten blocks were performed with plain lidocaine and nine with adrenalinated lidocaine.

The three injections were administered to each subject within four minutes. No significant cardio-pulmonary or hemodynamic effect, as indicated through respiratory rate, heart rate or blood pressure, was recorded during block execution. Two dogs developed a Horner’s syndrome that had no visible impact on their individual PK profiles, and resolved in 130 minutes following the reversal of anesthesia.

**Fig 2:**
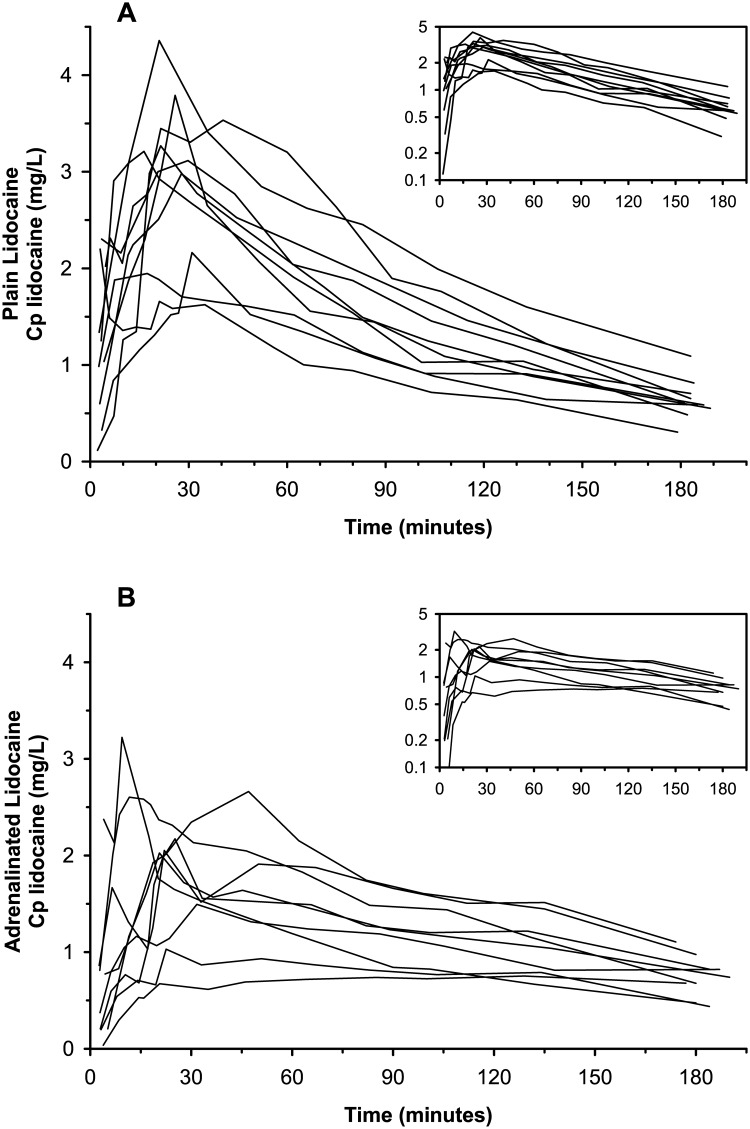
Individual time-courses of plasma lidocaine concentrations in dogs. (A) Paravertebral dosing of 6 mg/kg plain lidocaine formulation. (B) Paravertebral dosing of 6 mg/kg adrenalinated lidocaine formulation. The time-concentration data is delineated in linear coordinates (main graphs) and semilogarithmic coordinates (insert graphs).

The individual time-courses of plasma lidocaine concentration following administration of the plain and adrenalinated lidocaine formulations are shown in Fig 2. The elimination phase following C_max_ was visibly steeper for plain lidocaine than for adrenalinated lidocaine. In addition, the inter-individual variability was greater for adrenalinated lidocaine during the first 50 minutes following drug administration. But beyond 135 min following drug administration, the plasma lidocaine concentrations and terminal slopes of both treatment groups were strikingly similar (Fig 2).

### Model selection

**Fig 3:**
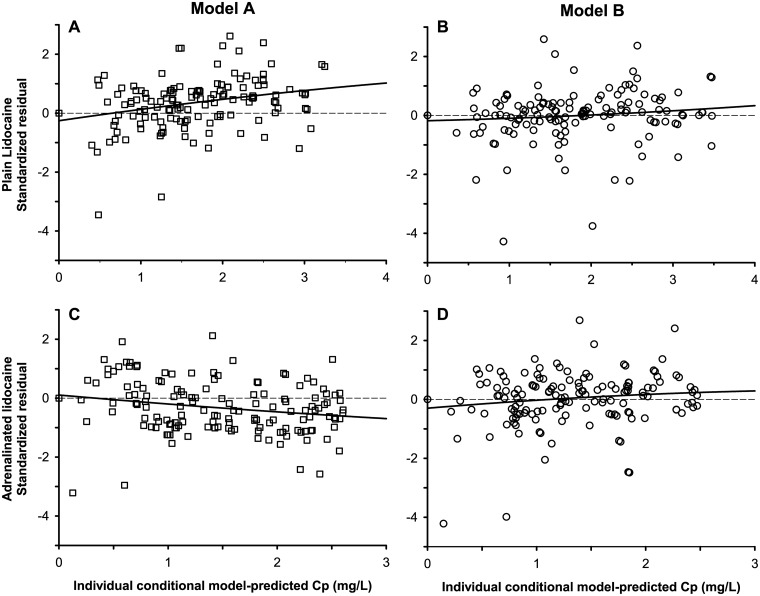
Lidocaine standardized residuals of the standard and alternative compartmental models *vs*. model-predicted plasma lidocaine concentration. (A) Plain lidocaine data predicted by Model A (i.e. one single first-order absorption rate per system). (B) Plain lidocaine data predicted by Model B (i.e. two asynchronous first-order absorption rates per system). (C) Adrenalinated lidocaine data predicted by Model A. (D) Adrenalinated lidocaine data predicted by Model B. A third degree polynomial curve (continuous line) has been added to highlight the trend in the residuals.

Model A had a lower AIC value than model B (-2859.34 vs. -2524.94, respectively), but the scattering of its standardized regression residuals as a function of predicted plasma concentrations showed departure from the abscissa for both lidocaine formulations (Fig 3A and 3C), indicating respectively that this model underestimated the higher plasma lidocaine concentrations of the plain lidocaine formulation, but overestimated the ones of the adrenalinated lidocaine formulation.

**Fig 4:**
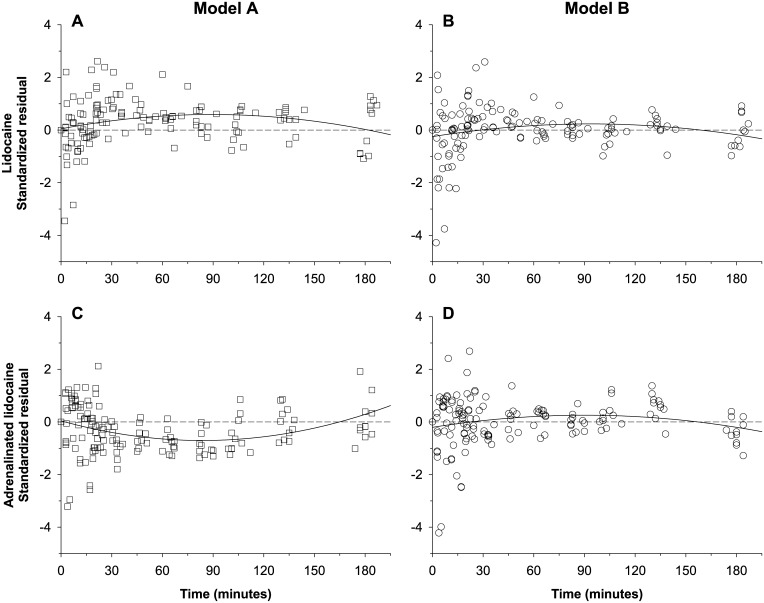
Lidocaine standardized residuals of the standard and alternative compartmental models *vs*. time following lidocaine administration. (A) Plain lidocaine data predicted by Model A (i.e. one first-order absorption rate per system). (B) Plain lidocaine data predicted by Model B (i.e. two asynchronous first-order absorption rates per system). (C) Adrenalinated lidocaine data predicted by Model A. (D) Adrenalinated lidocaine data predicted by Model B. A third degree polynomial curve (continuous line) has been added to highlight the trend in the residuals.

The plot of its standardized residuals as a function of time was curvilinear (Fig 4A and 4C) and showed large variability in the first 50 minutes of sampling as compared to model B (Fig 4B and 4D), indicating that Model A did not predict the absorption and peak phases of the kinetic profiles of both lidocaine formulations. Therefore, model B was selected for further refinement.

**Fig 5:**
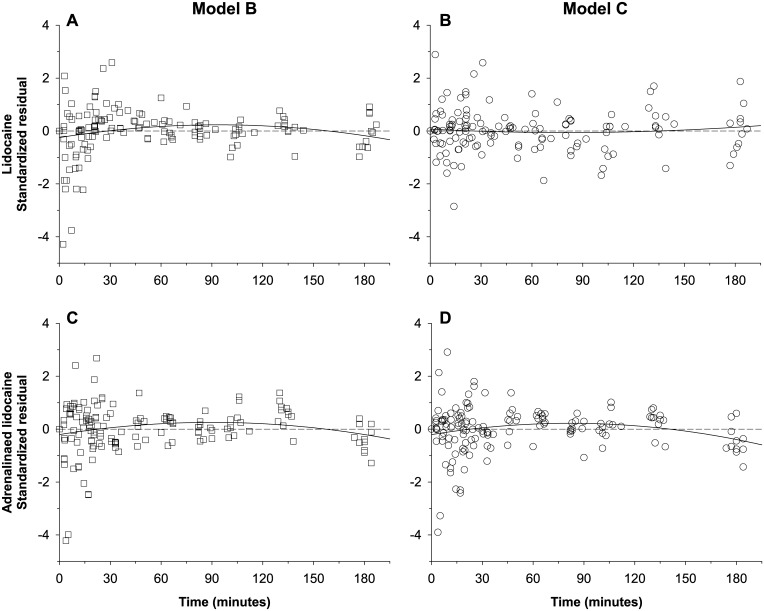
Lidocaine standardized residuals of the alternative and refined alternative compartmental models *vs*. time following lidocaine administration. (A) Plain lidocaine data predicted by Model B (i.e. with two asynchronous first-order absorption rates per system). (B) Plain lidocaine data predicted by Model C (i.e. with two time-constrained, asynchronous zero-order absorption rates per system). (C) Adrenalinated lidocaine data predicted by Model B. (D) Adrenalinated lidocaine data predicted by Model C. A third-degree polynomial curve (continuous line) has been added to help visualizing the trend in the residuals.

Model C had the smallest AIC value (-2913.48) of all three models and, compared with Model B (Fig 5A and 5C), standardized residuals as a function of time were closer to the abscissa, in particular during the first 50 min following administration of either lidocaine formulation (Fig 5B and 5D).

**Fig 6:**
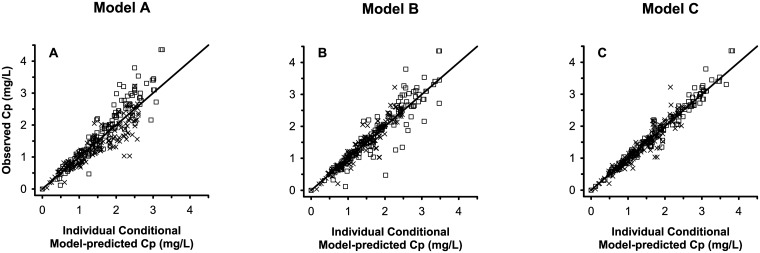
Observed *vs*. individual-conditional model-predicted plasma lidocaine concentrations following administration of plain or adrenalinated lidocaine in dogs. (A) Standard model (i.e. with one first-order absorption rate per system). (B) Alternative model (i.e. with two asynchronous first-order absorption rates per system). (C) Refined alternative model (i.e. with two time-constrained, asynchronous zero-order absorption rates per system). An identity line (*i*.*e*. Y = X) has been added to each plot as the expected relationship. Squares, plain lidocaine data. Cross marks, adrenalinated lidocaine data.

The scattering of observed *vs*. model-predicted plasma concentration data of Model C was closer to the identity line and was less dependent on concentration as compared to Models A and B (Fig 6C vs. 6A and 6B).

### Lidocaine absorption kinetics and adrenaline effects

**Fig 7:**
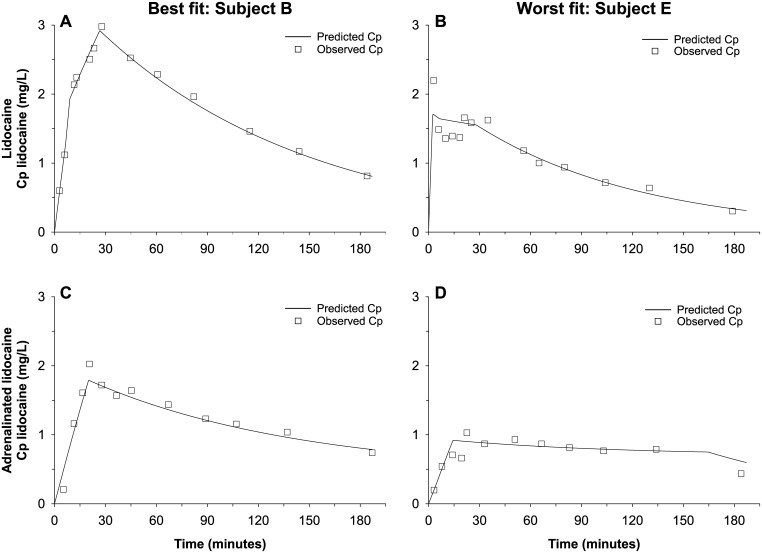
Best and worst fits to the individual time-courses of plasma lidocaine concentration in dogs. Predicted plasma lidocaine concentrations were generated by the refined alternative model (i.e. Model C, with two time-constrained, asynchronous zero-order drug absorption rates per system). (A) Best fit: Dog B, plain lidocaine data. (B) Worst fit: Dog E, plain lidocaine data. (C) Best fit: Dog B, adrenalinated lidocaine data. (D) Worst fit: Dog E, adrenalinated lidocaine data. Cp, plasma drug concentration.

Model C was selected to elucidate the absorption kinetics of lidocaine and examine the effect of adrenaline. Fig 7 presents the time-course of observed and model-predicted plasma lidocaine concentrations for two subjects, which respectively show the best (dog B) and worst (dog E) goodness-of-fit.

The estimated population pharmacokinetic parameters of lidocaine in dogs dosed with the plain and adrenalinated lidocaine formulations are detailed in Table 1. Adrenaline significantly decreased AUC_0-tlast_ by approximatively 23%, C_max_ and τ by 60%, k_01_ by 50% and k_02_ by 90% (p < 0.05 in all cases). It also significantly increased Dur_1_ by 90%, and Dur_2_ by and 1300% (p < 0.02), but did not significantly affect the estimated t_max_ and f of the tested formulations (p > 0.05).

**Table 1:** Estimated pharmacokinetic parameters of paravertebrally injected lidocaine in dogs, and statistical comparison of plain and adrenalinated formulations.

Parameter	Unit	Source	Plain formulation	Both formulations	Adrenalinated formulation	*P* value
**CL/F**	L·min^-1^·kg^-1^	Model		0.017 (0.005)		-
**V/F**	L·kg^-1^	Model		1.850 (0.580)		-
**λ**_**z**_	min^-1^	Model		0.009 (0.004)		-
**t**_**½**_	min	Model		75.0 (32.3)		-
**AUC**_**0-tlast**_	min·mg·L^-1^	Data	281.8 (83.2)		215.9 (69.1)	**0.0321**
**C**_**max**_	mg·L^-1^	Data	2.944 (0.818)		1.847 (0.636)	**< 0.0001**
**t**_**max**_	min	Data	19.72 (9.99)		13.53 (7.74)	0.1556
**Dur**_**1**_	min	Model	5.77 (3.24)		11.30 (7.05)	**0.0197**
**Dur**_**2**_	min	Model	18.10 (4.87)		254.00 (129.00)	**< 0.0001**
**τ**	min	Model	6.52 (0.02)		3.87 (0.03)	**< 0.0001**
**k**_**01**_	mg·min^-1^	Model	0.075 (0.055)		0.037 (0.029)	**0.0463**
**k**_**02**_	mg·min^-1^	Model	0.013 (0.008)		0.001 (0.001)	**< 0.0001**
**f**	mg·mg^-1^	Model	0.56 (0.22)		0.54 (0.15)	0.3640

Estimated pharmacokinetic parameters are expressed as log-normal means (log-normal SD), and were derived directly from the time-concentration data, or as the result of analysis with the refined alternative pharmacokinetic model (i.e. Model C, with two time-constrained, asynchronous zero-order absorption rates per system). AUC_0-tlast_, area under the plasma concentration curve between the time of dosing and the time of last measurable plasma concentration; CL/F, apparent total plasma clearance; C_max_, maximum plasma concentration; Dur_1_, duration of the early absorption phase; Dur_2_, duration of the late absorption phase; f, Dose fraction absorbed during the early absorption phase; k_01_, zero-order rate of the early absorption phase; k_02_, zero-order rate of the late absorption phase; λ_z_, terminal disposition rate constant; t½, terminal half-life; τ, delay to the onset of the late absorption phase; t_max_, time of maximum plasma concentration; V/F, apparent distribution volume.

## Discussion

The results reported herein support our hypothesis regarding the ability of our alternative compartmental model at accurately measuring the lowering effect of adrenaline on the absorption rate of perineurally-administered lidocaine in dogs. The estimated absorption kinetic parameters reported in Table 1 are consistent with a slower, prolonged systemic uptake of the adrenalinated local anesthetic from the injection sites.

The absorption phase of lidocaine was visibly biphasic for both dosing formulations, with a first fraction f that was absorbed at a faster rate, and a second dose fraction (1 − f) that was absorbed at a slower rate. In the case of plain lidocaine, both absorption rates were faster than the elimination rate of lidocaine, which induced a shoulder in the absorption phase of the kinetic profile. But in the case of adrenalinated lidocaine, the 2^nd^ absorption rate was slower than the elimination rate of the drug (*i*.*e*. flip-flop kinetics), which induced either a plateau or a shallow decreasing slope after C_max_. This and the visible trends in the standardized regression residuals (Fig 3A and 3C and Fig 4A and 4C) warranted the rejection of the “null hypothesis” Model A despite its lower AIC value as compared to model B. Other investigators already have reported such biphasic absorption kinetics for lidocaine and other local anaesthetics in human beings [22–26]. The mechanistic basis of this model cannot be ascertained from our data, but at least 4 options are possible. First, the fast absorption compartment may represent the dissolved drug molecules present at the site of injection receiving the highest blood supply, and the slow absorption compartment the drug molecules in a region of the injection site that receives a lower level of blood perfusion [25]. Second and consistent with our observations of dyes injected in the paravertebral brachial plexus of canine cadavers, the fast absorption compartment may represent an anatomical site proximal to a diffusion obstacle (*i*.*e*. fascia or fatty tissue), and the slow absorption compartment may represent the site distal to it. Third, the fast and slow absorption components may respectively reflect the blood *vs*. lymphatic transit pathways from the injection site to the systemic bloodstream. This possibility is receiving increased attention in parallel with the current development of therapeutic proteins and other large drug molecules [27]. Fourth, the dual fast/slow absorption may reflect a process of precipitation of this lipophilic drug at the site of injection. This phenomenon may result from the resorption of the aqueous drug solvent and the pH shift caused by the buffered interstitial fluids, which may increase the injection site drug concentration to the point of saturation [28].

The regression residuals of model B (*i*.*e*. with asynchronous fast and slow first-order absorption rate constants) scattered randomly over the abscissa of model-predicted concentration (Fig 3B and 3D), but an N-shaped trend was detected for the residuals of the first 50 minutes after drug injection, especially with plain lidocaine administration (Fig 4B and 4D). Therefore, this model misspecification suggestive of an atypical drug absorption process warranted the refinement of model B [29].

One possible modification is to replace the dual first-order, asynchronous absorption rates with two zero-order, time-constrained absorption rates, a strategy tried for the PK modeling of the epidural block with levobupivacaine [15]. Although these investigators found that the goodness-of-fit of this alternative model was lower than their original model, other authors have found it useful to fit the i.m. absorption kinetics of several other aqueous drug products such as trovafloxacin [30], pralidoxime [31], or recombinant human luteinizing hormone [32]. Another possible modification is to use two asynchronous inverse-gaussian absorption processes, a strategy that successfully quantified the effect of adrenaline on the uptake of ropivacaine in a thoracic paravertebral or a femoral nerve block [25,26]. In the current study, we improved our model by using the dual zero-order, time-constrained, asynchronous absorption rates. This alternative Model C had a markedly lower AIC value as compared to models A and B, an improved overall goodness-of-fit (Fig 6C vs. 6A and 6B), and random scattering of residuals over the abscissas, both as a function of model-predicted lidocaine concentration and as a function of time (Figs 4B and 4D and 5B and 5D).

In contrast with the studies cited above [15,25,26], the time-course of plasma lidocaine concentration had quasi-straight absorption phase slopes with abrupt slope changes (Fig 7), a shape that resembles a short stepwise i.v. infusion and is suggestive of at least two zero-order absorption processes [29]. This hypothesis was confirmed by comparing the AIC for models B and C. Our study design cannot elucidate the physiological determinants of a zero-order absorption profile of the tested aqueous solutions of lidocaine hydrochloride, but such atypical absorption behavior may be associated with the lymphatic uptake of precipitated drug molecules [27], or their dissolution by the rate-limiting turnover of interstitial fluid, resulting in pseudo zero-order absorption [30]. Further research is needed to confirm this hypothesized mechanism for lidocaine hydrochloride [33].

In contrast with levobupivacaine [15] and ropivacaine in humans [25], the zero-order absorption rates (k_01_ and k_02_) of lidocaine, their respective durations (Dur_1_ and Dur_2_), and the lag time for the onset of the second absorption rate significantly differed between formulations (*P* < 0.05). These changes in the absorption kinetics of lidocaine resulted in a significantly lower C_max_, hence a lower toxicity potential [34]. Interestingly, no significant difference was found for the partition coefficient f, a result that differs from the reported effect of adrenaline on the caudal epidural absorption kinetics of levobupivacaine in humans [15], and of ropivacaine injected paravertebrally in the thorax region [25]. In both studies, adrenaline significantly decreased the dose fraction that was absorbed from the fast-absorption compartment.

One limitation to our study is the lack of a study arm where each lidocaine solution was administered intravenously to the dogs. This precluded the estimation of the disposition pharmacokinetic parameters CL and V, of its terminal elimination rate, and of the absolute bioavailability F. But using one or more i.v. study arms in the same dogs may decrease the intrinsic clearance of lidocaine [35], and therefore hinder the elucidation of the effects of adrenaline on its absorption kinetics. Still, the disposition pharmacokinetic parameters estimated in this study are comparable to those of previous studies: one reported a CL of 0.04 L·min^-1^·kg^-1^ and a V of 1.44 L·kg^-1^ after a lidocaine i.v. bolus [18], and another reported a 70 min terminal half-life after a single intraperitoneal and incisional administration of lidocaine in dogs, with a calculated average CL/F of 0.056 L·min^-1^·kg^-1^ [36].

Additional limitations of this study include the small number of subjects, and the narrow blood-sampling window, especially for the adrenalinated lidocaine administration. But the latter problem was circumvented by assuming that, at the dose and dosing interval used here, adrenaline has negligible effects on the disposition kinetics of lidocaine. This enables the use of a compartmental model where the PK systems for plain and adrenalinated lidocaine share identical CL/F and V/F, and therefore the terminal slope can be detected even if one sampling time of the adrenalinated lidocaine formulation occurred in the elimination phase.

The tested drugs were administered under isoflurane general anesthesia, which was shown to alter lidocaine’s pharmacokinetic parameters in cats [11]. However, anesthesia was stopped as soon as the last block injection was completed, and therefore most blood samples were taken on ambulatory dogs.

Finally, with respect to the dosing schedule, differences in the early post-C_max_ portion of the time-course of plasma lidocaine concentrations after dosing adrenalinated lidocaine on single *vs*. multiple paravertebral injection sites have been reported [37]. In our study, three injections were administered to each subject within a maximum window of four minutes, and the recorded slope of plasma drug concentrations did not change following each block injection. We therefore chose to consider our dosage regimen as a single administration.

## Conclusions

This crossover comparison of the pharmacokinetics of plain and adrenalinated lidocaine solutions in dogs subjected to a paravertebral brachial plexus block quantified the slowing effect of adrenaline on the systemic absorption rate of lidocaine. But most importantly, it revealed a previously unreported atypical absorption pattern for this local anaesthetic drug, which is independent of the presence of adrenaline. The physiological and/or physicochemical determinants of this sequential, time-constrained, zero-order absorption kinetics remain to be elucidated, but may explain our limited ability to predict the depth and duration of local anesthesia when the local anaesthetic is administered at different doses or in repeated dosings.

## Supporting Information

S1 File Lidocaine time-concentration data in dogs dosed paravertebrally with plain and adrenalinated lidocaine formulations. Subject#, subject number; ID, identification; Formulation, lidocaine formulation used; Time (min), time following the initiation of the 1st injection; Cp (mg/L), plasma lidocaine concentration (mg/L).
(XLSX)
